# Effect of Osmotic Pretreatment Combined with Vacuum Impregnation or High Pressure on the Water Diffusion Coefficients of Convection Drying: Case Study on Apples

**DOI:** 10.3390/foods10112605

**Published:** 2021-10-28

**Authors:** Monika Janowicz, Agnieszka Ciurzyńska, Andrzej Lenart

**Affiliations:** Department of Food Engineering and Process Management, Institute of Food Sciences, Warsaw University of Life Sciences, SGGW, 159c Nowoursynowska St., 02-776 Warsaw, Poland; monika_janowicz@sggw.edu.pl (M.J.); andrzej_lenart@sggw.edu.pl (A.L.)

**Keywords:** diffusion coefficient, drying convection, pressure pretreatment, osmotic dehydration, apples

## Abstract

The paper presents water diffusion coefficients as providing a significant contribution to the creation of a comprehensive database and knowledge of weight variation during the drying process of raw plant materials that is used for modelling the technological process and designing innovative products. Dehydration is one of the most widely used methods for improving the stability and durability of fruit and vegetables because it reduces water activity and microbial activity, and minimises the physical and chemical changes during storage. The considerable impact of pressure on heat exchange and weight during the convection drying process of osmotically pretreated apples is demonstrated. The course of the drying curves and the drying rate is determined by the use of pressures of 0.02 and 500 MPa. Varied pressure applied during osmotic impregnation significantly influences the value of the diffusion coefficient: the average determined for the entire course of the drying curve and the average determined in the intervals of the reduced water content. The lowest values of the average water diffusion coefficient are obtained for apples preboiled under overpressure conditions and, at the same time, the determined diffusion coefficients in the water content are characterised on the drying curve by a clearly decreasing course until the reduced water content reaches approximately 0.2.

## 1. Introduction

To determine the quality of foods of plant origin, especially processed as a result of water removal, the following characteristics are most often used: structure, including density, porosity and consistency, optical—colour, general appearance, sensory—taste, smell, rehydration ability and nutritional value. They depend on many process factors, but, most of all, they depend on the properties of the raw material [1,2]. The attractiveness of dried food products based on the raw materials of plant origin depends on their quality. The raw materials of an appropriate quality allow products to be obtained that meet the expectations of a prospective consumer. Changes occurring in the plant material during drying may be modified by controlling the process parameters. Significant changes in the properties of the raw material are prevented by appropriate preliminary treatment. Various types of pretreatment are applied prior to drying in order to later reduce the undesirable effects of temperature gradients in the tissue structure during convection drying. Fruit and vegetables preserved by the osmotic–convection method retain the colour, smell and taste almost unchanged, despite being treated for a long time at an increased temperature. The unchanged colour of the material results from the inhibition of the activity of polyphenyl oxidase, which causes the enzymatic browning process. The preservation of the smell, and in many cases its intensification compared to the material obtained only by convection, is associated with the formation of a layer of osmotic substance on the surface of the plant material. This hinders the movement of moisture and, thus, also the possibility of a loss of aromatic compounds. The osmotic treatment removes some salt and organic acids from the raw material. As a result, there is a change in the ratio of sugar to acids, affecting the organoleptic assessment of the finished product [3,4,5,6,7,8]. In addition to the many advantages mentioned above, the material treated with this method is characterised by a porous structure, smooth surface, and almost unchanged shape and size, which affects the visual impression of the potential consumer and facilitates its rehydration in water [9,10,11].

Conventional drying techniques and the possibility of combining traditional drying with unconventional methods of food preservation, create new opportunities for the food industry. The aim of these techniques is, nowadays, to meet the increasing demands of the consumer, as well as to provide safe, comfortable products characterised by the repeatability and functionality of properties, as well as high nutritional value. The search for new methods of fixation causes new process parameters to be tested, resulting in combined methods being used in order to give favourable results. Therefore, food is preserved on an industrial scale by both temperature changing and non-thermal methods. High hydrostatic pressure (HHP) techniques belong to the group of unconventional methods of ensuring adequate food quality. The use of the HHP treatment allows not only to extend the shelf life of food by eliminating pathogenic microorganisms but also to change its properties, thus giving the possibility of creating new food products. The high hydrostatic pressure technology is a processing method characterised by its short time, high efficiency, low energy consumption and simple operation process, which has been recognised as an environmentally friendly method by the American Food and Drug Administration [12]. The use of high hydrostatic pressure treatment significantly improves the conditions of heat and weight exchange in diffusion processes. This happens regardless of the diversity of plant tissue at which the action is directed and regardless of the combination of the parameters used [13,14,15]. The previous work has mainly focused on the kinetics and modelling of the HHP technique [16], its drying efficiency and water permeation rate, and the microstructure changes after the HHP pretreatment. The combination of osmotic dehydration, treatment under varied pressure and drying makes it interesting to understand the conditions of weight exchange occurring during the removal of water from the plant tissue.

Water diffusion coefficients constitute an indispensable contribution to the creation of a comprehensive database of weight variation during the drying process of plant raw materials. Knowledge in this area is necessary for modelling technological processes and designing innovative products. A number of technological processes in the food industry are based on the movement of specific components at the boundary of a solid–liquid or inside a sample. Dehydration, due to both osmotic pressure and evaporation as a result of drying, is one of the most commonly used methods of improving the stability and durability of fruit and vegetables because it reduces the water activity, reduces microbial activity and minimises physical and chemical changes during storage [17,18]. The authors’ own research [19] and many other research projects [13,20,21,22,23,24,25,26] on the impact of osmotic dehydration on the convection drying process of apples have confirmed that the heat and weight exchange mechanism in this process resembles diffusion. The results of the research on drying pre-dehydrated apples under varied process parameters (a type of osmotic substance, pressure) under convection have proven a decreasing rate of water removal along with reaching the equilibrium water content in the sample. The efficiency rate of water removal depends on the factors directly affecting the diffusion conditions. The extent of changes is influenced by the parameters of the dewatering process as well as the changes in the composition and internal structure occurring in the dehydrated matter.

The vast majority of existing scientific publications have focused on the effect of osmotic dehydration performed at atmospheric pressure, a vacuum or high pressure, on the subsequent drying process. However, the presented research is the only one that has studied the osmotic dehydration process under a wide range of pressures—both low and high values in different variants. In this manner, the article can serve as a source of valuable knowledge so important before process optimization.

The aim of the research is to explain the influence of the pressure used during pretreatment on the course of the convection drying process, and the change in the water diffusion coefficient resulting from the use of varied pressure during the osmotic treatment. The work included the use of reduced and elevated pressure relative to atmospheric pressure. At the same time an attempt was made to determine the water diffusion coefficient during the convective drying of apples.

## 2. Materials and Methods

### 2.1. Characteristics of Raw Materials

The test samples comprised “Idared” variety apples stored in a cold store at a temperature of +5–+ 8 °C at an air humidity of 80–90%. The variety was chosen for the research purpose because during the long availability period in the market, they keep concise and strong structure. They also constitute a model plant tissue with a porous structure, thanks to which they store various substances well.

**Figure 1:**
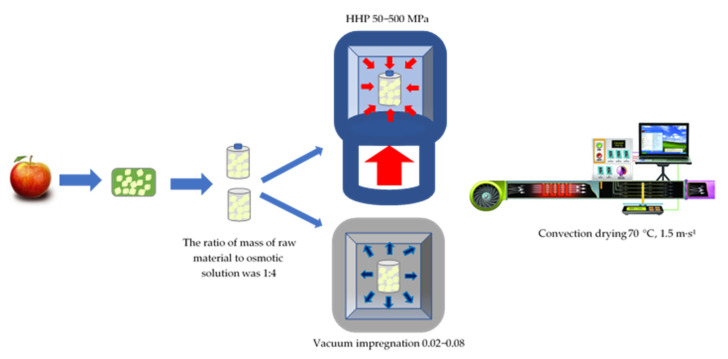
The diagram of the conducted experiments.

Apples were washed, peeled and cut into cubes (10 mm) (Figure 1). The chopped material was immersed in a 0.5% citric acid solution to protect against enzymatic browning reactions, then dried on a filter paper. The apple cubes prepared in this way underwent the technological treatment using osmotic dehydration in a sucrose solution (61.5°Bx) at a pressure ranging from 0.02 to 500 MPa, followed by convection drying.

Vacuum impregnation under varied pressure was carried out in a sucrose solution characterised by the water activity of 0.9. The ratio of raw material weight to osmotic solution was 1:4. The process time under varied pressure conditions was 180 min. The whole of the range of pressure in vacuum impregnation was 0.02–0.08, the apples were dehydrated throughout the process (180 min) under the pressure and at the temperature pre-set at the level of 25 ± 1 °C. The vacuum impregnation process was carried out in the laboratory chamber of the SPT-200 vacuum dryer (ZUT Colector, Kraków, Poland).

Pretreatment of the apples with high hydrostatic pressures was carried out using a high-pressure chamber owned by the Institute of High Pressure Physics (IWC) in Warsaw, also known as “Unipress”. Pretreatment of samples with the HHP was carried out in two stages: the first stage, obtaining the initial pressure of 80–150 MPa, and the second stage, obtaining the desired pressure (50, 100, 200, 500 MPa), holding for 5 min, and the remaining part of the osmotic dehydration was carried out under the atmospheric pressure of 0.1 MPa. The temperature of the samples during pressure, depending on the applied pressure, ranged from 23–27 °C and was higher corresponding to higher pressure.

The convection drying of apples was carried out in a laboratory dryer with forced air flow. The raw material was laid on metal nets in a single layer and dried at a temperature of about 70 °C with a drying air velocity of 1.5 m∙s^−1^. Drying was carried out until a constant weight was obtained under the given drying conditions for 20 min of the process. The weight loss was recorded continuously using the Axis electronic scale by Radwag AR-2000 with an accuracy of 0.1 g and “Measurement” software in the DOS (disk operating system) system. Water content in the tested sample at each stage of treatment was determined on the basis of the sample weight and the dry substance weight obtained by the drying method according to the AOAC [27] in vacuum dryer (Memmert VO400, Schwabach, Germany) (10 mPa, 70 °C, for 24 h).

The calculations were performed in triplicate for all technological operations.

### 2.2. Changes in the Structure of Apples

Changes in the structure of apples resulting from the applied technological procedures were determined on the basis of the analysis of photos taken at the Analytical Centre of the Warsaw University of Life Sciences, using the FEI Quanta 200 ESEM scanning electron microscope with the EDS micro-analyser and digital image recording. Observations were made without pretreatment of the samples at a pressure of 100–133 Pa, with an accelerating voltage of 25 or 30 kV. Equipping the microscope with a Peltier device for the purpose of controlling the temperature of samples allows for the examination of wet samples.

Calculation of water loss (*WL*) and the increase in dry matter (*SG*) [28]

(1) $$WL=\frac{\left(m_{o}-m_{i.d.m}\right)-\left(m_{1}-m_{d.m.}\right)}{m_{o}}*100\%$$

(2) $$SG=\frac{\left(m_{d.m.}-m_{i.d.m}\right)}{m_{o}}*100\%$$

where:

$m_{o}$—weight of apples before the dehydration process (g),

$m_{o1}$—weight of apples after the dehydration process (g),

$m_{i.d.m}$—the initial dry matter content in apples (g),

$m_{d.m.}$—dry matter content in apples after the osmotic dehydration process (g).

### 2.3. Mathematical and Statistical Analysis of Research Results

The mathematical elaboration of the results was performed using Excel and TableCurve2D software that allows an equation to be found describing data by searching for solutions from among a wide range of models. The statistical analysis of results was conducted using the analysis of variance based on the ANOVA (the one-way analysis of variance) summary table (StatSoft-Statistica 13.1. Inc., Tulusa, OK, USA). Tests were applied to verify the assumption of homogeneity of single- and multi-factor variance (adopted level of significance, *p* = 0.05). If many dependent and accompanying variables occurred in the statistical analysis, tests were presented for each dependent variable and accompanying variable. The least significant difference (LSD) test was carried out to compare the study results and correlations between them.

## 3. Results

### 3.1. Convection Drying of Osmotically Dehydrated Apples under Varied Pressure Conditions

**Figure 2:**
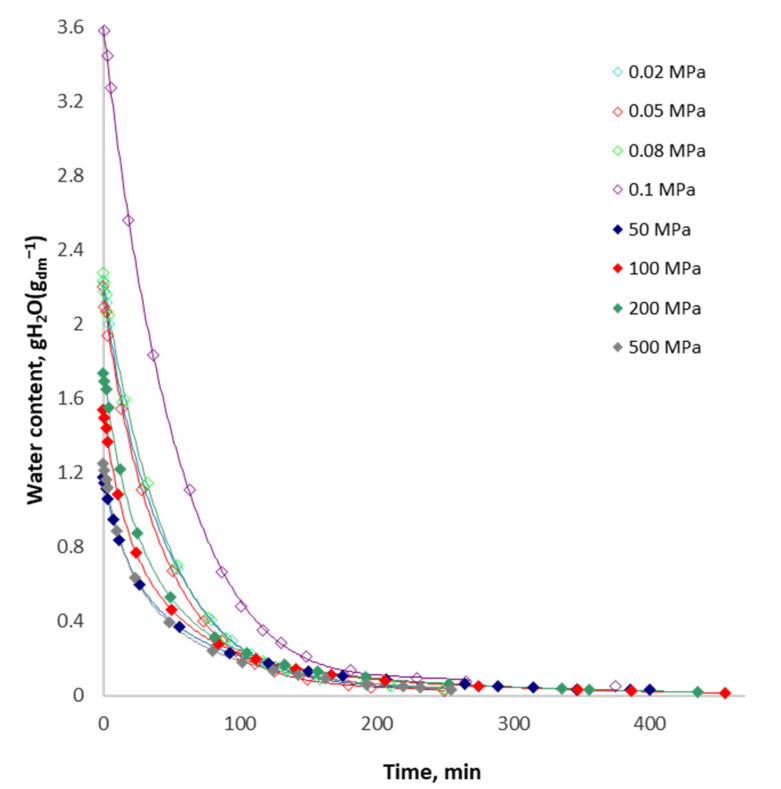
The course of the drying curves of apples initially osmotically dehydrated under varied pressure.

Figure 2 shows the course of drying curves for apples osmotically impregnated using varied pressure. Changes in the initial water content resulted from the osmotic impregnation of apples in the sucrose solution. The osmotic dehydration affected the course of the process by changing its mechanism and the value of the diffusion coefficient of water in the sample. It was found that the use of osmotic dehydration under varied pressures influenced the achievement of a different level of dehydration and, thus, a different value of the initial water content after osmotic dehydration of the samples.

In addition, the pressure under which the pretreatment process takes place affects the final equilibrium water content of apples and the time of the drying process, which can be better seen in Table 1. As a result of the convection drying of apples vacuum impregnated under varied pressure (0.02–500 MPa), droughts with significantly different final equilibrium water content were obtained after significantly different drying times. The final equilibrium water content ranged from 0.015 to 0.050 g H_2_O∙(g d.m.)^−1^, which corresponds with the moisture range of 1.5–4.8%. At the same time, the drying time for apples ranged from 250 to 455 min depending on whether vacuum, atmospheric pressure or HHP was used for the osmotic dehydration, which was confirmed by setting three homogeneous groups (Table 1).

It was found that the use of the pressure above atmospheric pressure (50–200 MPa) during the vacuum impregnation significantly extended the time of convection drying to obtain the equilibrium water content in the dried apples at a temperature of 70 °C with an air flow of 1.5 m∙s^−1^. At the same time, the droughts after overpressure pretreatment were characterised by a significantly lower final equilibrium water content in the entire range of applied overpressure conditions (50–500 MPa) (Table 1).

An inverse trend in the drying time was observed in the case of the samples vacuum impregnated under lower than atmospheric pressure as compared to the treatment under atmospheric pressure. The application of osmotic pretreatment under lower than atmospheric pressure significantly reduced the time of convection drying to obtain the equilibrium water content (Table 1).

The statistical analysis of the obtained results confirmed a significant reduction in the equilibrium water content for dried apples vacuum impregnated under lower than atmospheric pressure and a significant reduction in drying time compared to apples osmotically dehydrated under atmospheric pressure. The analysis of the results obtained for the vacuum impregnation only in the range of reduced pressure (0.02–0.08 MPa) did not prove the impact of the applied osmotic dehydration on the parameters under consideration (final equilibrium water content and drying time).

The statistical effect of the applied HHP on the final equilibrium water content in the dried apples and drying time was also demonstrated, both in relation to the apples subjected to osmotic treatment under atmospheric pressure and in the range of 50–200 MPa. A significant effect on the extension of the process time was proven for the overpressure range of 50–200 MPa. In the case of the application of the osmotic treatment under the pressure of 500 MPa, the drying time of the apples was significantly shortened as compared to the other conditions at issue, such as overpressure and atmospheric pressure.

**Figure 3:**
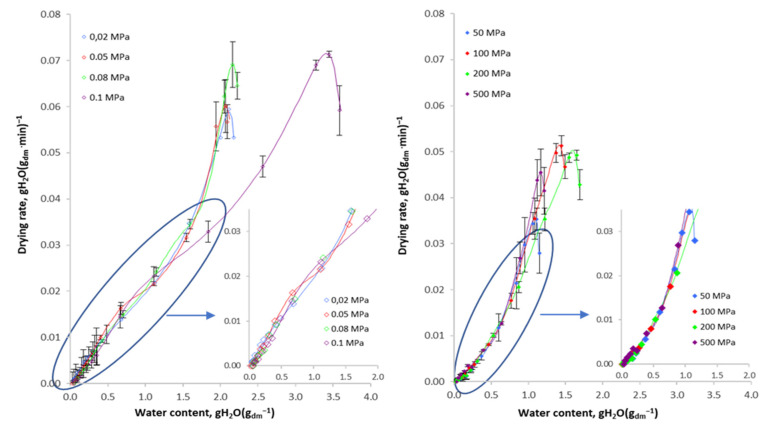
The course of the drying efficiency rate curves of apples initially osmotically dehydrated under varied pressure.

**Figure 4:**
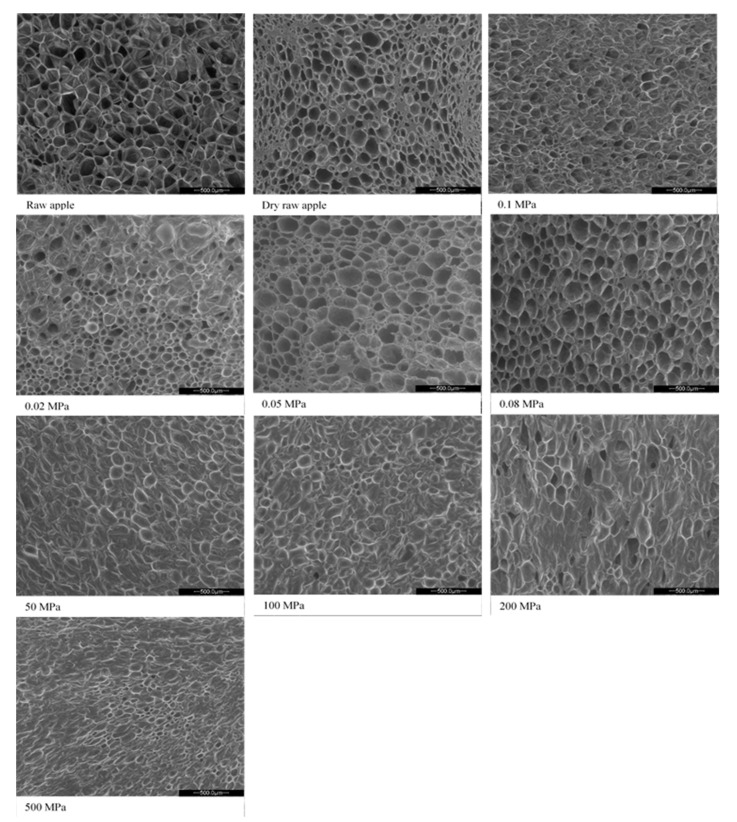
Structure of convectional dried apples non-dehydrated and osmotically dehydrated under lower than atmospheric pressure and higher than atmospheric pressure.

Figure 3 shows the course of the drying efficiency rate curves of osmotically dehydrated apples under varied pressure. It was found that the drying of apples osmotically dehydrated under atmospheric pressure was characterised by the lowest drying rates at a certain water content as compared to the dehydrated sample both under and over the atmospheric pressure and higher than the atmospheric one. The analysis of the curves also allowed a change to be observed in the shape of the drying efficiency rate curves depending on whether the osmotic dehydration process was carried out under overpressure. The change in the course is clearly visible in the period of decreasing the drying efficiency rate below the water content in the sample at the level below 0.03 g H_2_O∙(g d.m.)^−1^, which may be seen in the enlargements attached to the drawing (Figure 3). When defining the course of the curves according to the type of dried material, it may be assumed that the structure of apples obtained as a result of vacuum impregnation under lower than atmospheric pressure corresponds to the type of tissue with capillary–porous properties of a complex structure. The shape of the drying curves of osmotically dehydrated apples under higher than atmospheric pressure (50–200 MPa) characterises the sample with a clay-like structure during the drying process. As a result of such structural changes in the sample, not only the weight and heat exchange that determine the course of the process are changed but also the characteristics of ready-made droughts (structure, mechanical properties). Changes of this nature may be explained by the diffusivity of apple tissue. As a result of the two-way weight exchange phenomenon that occurs as a result of changes in the osmotic pressure in the diffusion system, an apple tissue, a hypertonic substance, cell sap is only superficially replaced with a sucrose solution, the presence of which in the material is clearly visible in the photos of the structure of the dried material compared to fresh material and not osmotically dehydrated (Figure 4). At the same time, the weight exchange causes a significant reduction in the initial water content in the material, regardless of the pressure applied, as compared to the sample dewatered under atmospheric pressure (≈3.6 g H_2_O∙(g d.m.)^−1^) (Figure 2).

It was shown that as a result of diffusive mass movement, water loss (*WL*) was greater than the increase in dry matter weight (*SG*) (Table 2).

It was also found that in the case of drying apples osmotically dehydrated under lower than atmospheric pressure, a reduction in the initial water content was recorded, which resulted in a significant reduction in the drying time to obtain the equilibrium water content. The tendency was also confirmed in the case of using osmotic dehydration under pressure higher than atmospheric at the level of 500 MPa, while for higher than atmospheric pressure in the range of 50–200 MPa the relationship was reversed (Table 1).

### 3.2. An Attempt to Determine the Water Diffusion Coefficient during the Drying of Osmotically Dehydrated Apples under Varied Pressure

The diversity of considerations on the drying process resulted in the necessity to choose a parameter that would describe the drying process in an unambiguous manner and independent of the method of weight variation at a given moment. Therefore, the mechanisms, regardless of the technical mode, are described by the effective diffusion coefficient. The knowledge of this parameter allows for an appropriate interpretation of the drying process, as well as for a legal description of it in terms of mathematics. In order to determine the diffusivity not only during drying but also in other weight variation processes (rehydration, extraction, osmotic dehydration, etc.), the Fick equation is used, the analytical solution of which, taking into account the geometry of the sample and under certain assumptions, allows simplification of the interpretation of the weight variation process. The correct determination of water diffusivity during drying requires the assumption that the water content on the surface of the material is in equilibrium with the air humidity. It should also be assumed that the drying conditions and diffusivity remain unchanged, regardless of the variability of the water content and temperature in the dried sample.

Based on the assumptions of the second Fick’s law, assuming the initial and boundary conditions of the first type and taking into account the shape of the initial material (infinite plate) in the equation, and making the appropriate simplification to the form of Equation (3), the water diffusion coefficient was calculated as described below. The proposed mathematical description of the second drying period allowed the determination of the water diffusion coefficient (*D_ff_*) using the regression method for each measurement series by determining the constant k. The coefficient *D_ff_* was determined from the dependence (3) in the entire tested drying range, i.e., within the range of changes in the relative water content (*MR*) from 1 to 0.0025. The range of variability of the relative water content (*MR*) was divided into ranges from 1 to 0.1, every 0.05, and from 0.05 to 0.005, every 0.005. Depending on the experiment carried out, in some cases two adjacent compartments were joined, which facilitated the determination of the water diffusion coefficient for the average value of the relative water content in a given compartment.

Drying curves obtained as a result of experiments (Figure 2) are exponential and are described by the following equation formula

(3) $$\mathrm{MR}=\frac{u_{\tau -u_{r}}}{u_{0-u_{r}}}=\Psi *e^{-K\tau }$$

where:

$\Psi =\frac{8}{\pi^{2}}$—the aspect ratio of the infinite plate of double-sided dried samples,

*u*_0_ = initial water content, gH_2_O∙(g d.m.)^−1^,

$u_{\tau }$ = temporary water content, gH_2_O∙(g d.m.)^−1^,

τ= time, min,

*K* = drying constant.

In order to perform a comparative analysis of convection drying curves, a correction factor A was introduced into the general equation describing the drying kinetics. This allowed differences in water content to be taken into account in osmotically dehydrated apples, which were also the initial water content in the convection drying process by modifying the equation to

(4) $$\mathrm{MR}=\left(\Psi -A\right)*e^{-K\tau }$$

where:

*A* = correction factor,

*MR* = relative water content.

After taking into account, in the above equation, the shape of the research input and the correction coefficient defined above as well as boundary conditions commonly used in the description of heat exchange and weight of the second drying period in convection conditions, a new dependence is obtained

(5) $$\mathrm{MR}=\left(\frac{8}{\pi^{2}}-A\right)*exp\left(\frac{\pi^{2}*D_{ff}}{2*l^{2}}*\tau \right)$$

where:

*D_ff_* = diffusion coefficient of water (m^2^∙s),

*l* = length (m).

The proposed mathematical description of the second drying period allows for the determination of the conventional diffusion coefficient of water. Using the regression method for each series of measurements, the constant *K* was determined according to the scheme

(6) $$\mathrm{MR}=\left(\frac{8}{\pi^{2}}-A\right)*exp\left(-K*\tau \right)$$

where:

$$K=\frac{\pi^{2}*D_{ff}}{2*l^{2}}$$

The mean values for the whole apple drying process determined in this way and calculated on the basis of the above dependences, indirectly allowed assessment of the influence of osmotic dehydration parameters on the convective drying kinetics.

**Figure 5:**
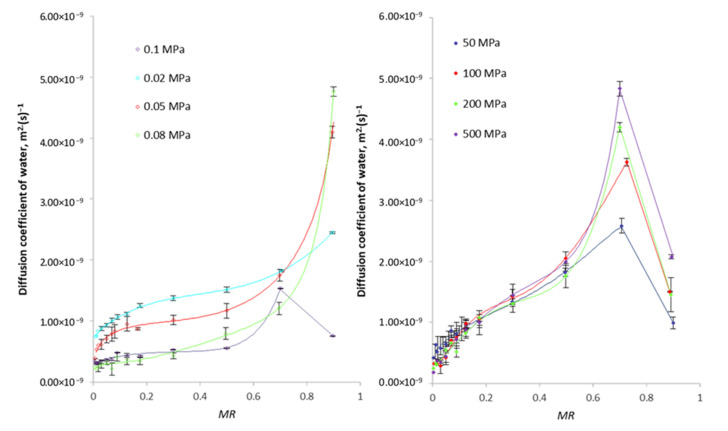
Change in the water diffusion coefficient during drying of apples pre-osmotically dehydrated under varied pressure.

Fruit subjected to varied pressure is characterised by a different value of the diffusion coefficients *D_ff_* as compared to the sample dewatered at atmospheric pressure, which may indicate a greater permeability of cell walls caused by pressure stress. As a result, it may facilitate mass exchange during pretreatment, which results in the penetration of the hypertonic solution into the apple tissue (Figure 4) and provides higher values of the weight and heat variation coefficient during convection drying (Figure 5).

It was found that the use of a hypertonic solution as a medium allowed the reduction in the initial water content in the plant tissue (osmotic dehydration) before drying, and, at the same time, increased the rate of weight movement during the drying process, which resulted in a higher value of the water diffusion coefficient calculated in individual ranges of the relative water content *MR*. It was observed that for the osmotically dehydrated sample under high hydrostatic pressure, the course of changes in water diffusion during drying is similar to the course of changes in the tissue of dehydrated fruit under atmospheric pressure. The tendency characteristic for the discussed courses of [29] is a significant increase in the water diffusion rate in the range of the relative water content from 0.9 to 0.7. In the case of osmotically dehydrated apples under negative pressure (0.02–0.08 MPa) in the entire range of changes in the relative water content, the diffusivity of apples’ tissue significantly decreases. The values of the water diffusion coefficient for *MR* values at the level of about 0.9 are significantly higher than those for apples with initially reduced content water under the conditions of using high-pressure osmotic tissue treatment (50–500 MPa). It was found that the tissue mobility to weight variation increased as a result of pressure-induced stress, regardless of its value and the presence of a hypertonic substance in the tissue, as evidenced not only by photos of the internal structure of drained and dried apples (Figure 4) but also the value of the dewatering efficiency coefficients defined as the ratio water loss (*WL*) and the increase in dry substance weight (*SG*) presented in Table 2.

## 4. Discussion

In order to ensure the appropriate quality and microbiological stability, dried vegetables should have a water content of 10–15%, and fruit, 20–25%. It may be assumed that thanks to the application of pretreatment under different pressures, it is possible to obtain droughts with a high degree of water (1.5–4.8%) removal and, therefore, more stability during storage (Table 1). The selected method and type of technological processes have an impact on the amount of water contained in the dried material and the course of pretreatment and drying. This is confirmed by the authors’ own and other researchers’ investigations on drying: osmotic dehydration in varied conditions and drying using various methods [2,7,8,19,30,31,32,33,34,35,36]. The research results clearly indicate that the optimisation of technological processes in order to obtain the appropriate product quality should be the result of the analysis of several factors including, among others, processing conditions, preparation methods and physical properties of the processed raw material, and their integration in order to increase the acceptance of the dried product. The methodology and results of this type of research may be used for developing new food products and optimising existing ready-to-eat dried products [2,8,19,23,24,31,37,38].

The degree of water removal during osmotic dehydration is determined by two-way diffusion weight movement, process parameters (osmotically dehydrated under lower than atmospheric pressure and higher than atmospheric pressure) and the internal structure of plant materials. The diffusion rate of water from any material with tissue structure depends on factors such as the temperature and osmotic concentration of the solution, the size and geometry of the sample, the ratio of the weight of the sample to the weight of the solution, and the degree of damage resulting from the osmotic dehydration pretreatment [6]. Scientific research confirms the influence of variable weight movement parameters (osmotically dehydrated under lower than atmospheric pressure and higher than atmospheric pressure) on the course of fruit and vegetable dehydration [4,5]. Osmotic treatment without varied pressure support has little effect on the modification of the structure of plant raw materials and their sensory characteristics, but the use of varied pressure in combination with the reduced water content prolongs the shelf life of products and improves their quality. As a result, the product prepared in this way may become a raw material in a different type of technological process at a later stage of processing [5,7,8,9,10]. The action of the vacuum causes the internal gas to expand and flow out of the product, which carries the water with it. During relaxation, gas is reduced and the external substance enters the pore structure. Vacuum dehydration allows control of the solution introduction into the pores of the fruit [31]. The mixture may contain active compounds, antimicrobial agents, substances used to control food stability, freshness, nutritional or flavour value, minerals and vitamins. These products can enlarge the functional food market.

Mathematical models describing the absorption of the osmotic substance as exemplified by calcium and iron ions were developed, allowing for the process to be carried out in a controlled manner [39,40,41]. The effect of high hydrostatic pressure on food ingredients causes a strong strengthening and intensification of the processes taking place during the treatment processes [19,24,37,38,42]. The influence of high pressure on hydrogen, ionic and hydrophobic (non-covalent) bonds is ambiguous. They may be destroyed or formed under the action of the HHP, depending on the volumetric changes in the system [19,37,43,44,45]. Therefore, proteins, nucleic acids and starches, the quaternary structures of which are formed using these bonds, are denatured, coagulated or gelatinised. It may be concluded that the most frequently mentioned effects of the use of non-thermal food preservation and high pressures is, above all, the destruction of microorganisms ensuring the appropriate quality of food. However, it should be remembered that the HHP also denatures proteins or is responsible for their structural modification. Enzymes may be both inactivated and activated by the HHP treatment, depending both on the pressure applied and on the type of enzyme. As a result of pressure, the properties of polymers also change [13,19,25,26,29,36,38,45,46].

Weight variation studies on the osmotic dehydration of mango under varied pressure (Sulistyawati et al.) [25] found that the factor determining the degree of water removal from plant tissue depended not only on the value of the applied pressure but also on fruit ripeness. They observed that after negative pressure had been applied, gas was removed from the pores of the tissue, and excess pressure increased its permeability, which explained the changes in the efficiency factor of osmotic dehydration. In the case of mangoes, Tedjo et al. [20] also found a greater increase in dry matter weight in relation to the loss of water from fruit tissue in terms of weight variation during osmotic dehydration under elevated pressure. However, for the process carried out under negative pressure, it was confirmed by Torres et al. [21]. In studies conducted with the use of bananas [22], tomatoes [24], peaches and apricots [38], it was found that in the case of tissues of this type, as a result of diffusive mass movement, water loss (*WL*) was greater than the increase in dry matter weight (*SG*), which was also confirmed in the study of apple tissue in the presented considerations.

The research clearly shows the effect of water removal, with the use of diffusion in the process of osmotic dehydration in hypertonic solutions supported by varied pressure (osmotic dehydrated under lower than atmospheric pressure and higher than atmospheric pressure), on the course of convective drying. The effect of this influence is a change in the drying course, especially in the drying time necessary to obtain an equilibrium water content in the dried apples and in the course of the drying efficiency rate curves. In particular, in the case of drying efficiency rate, characteristic curves are observed [29], which indicate significant changes in the weight variation process during diffusion in the drying process, resulting from varied processing conditions. Yucel et al. [13] also found that conventional drying of fruits and vegetables affected the physical and biochemical properties of the dried material, which in turn led to a significant tissue shrinkage and a change in structure, colour and taste. Therefore, the high pressure may be used for pretreatment prior to drying and, by its action, ensuring the permeability of the cell membrane and, thus, enhancing the weight variation, largely preserving the properties of the plant tissue. At the same time, the application of high pressure at the level of 100 MPa resulted in a significant reduction in the drying time of apples and carrots, while in the case of green peas, this effect was achieved by increasing the pretreatment temperature from 20 to 35 °C in addition to the HHP. In the studies of Yucel et al. [13], it was shown, however, that the higher the pressure used during the pretreatment, the shorter the drying time of the tested tissues of fruit and vegetables, which was not clearly confirmed in the studies carried out on “Idared” variety apples in this study. The results obtained by Zhang et al. [26] for strawberries suggest that the HHP pretreatment disrupted the integrity of the fruit tissue, which increased the efficiency of vacuum drying and freeze-drying. The HHP significantly increased the diffusivity of strawberry tissue, reducing the drying time by 9–24%. It seems that the lack of confirmation of the drying time reduction in the case of the research carried out on the tissue of apples of “Idared” variety apples, dehydrated in the pressure range of 50–200 MPa, may result from the osmotic pretreatment applied. The pressure both lowered and increased relative to atmospheric conditions during osmotic dehydration, in addition to changing the kinetics of weight movement and the structure of the product, but it depended on the elasticity and flexibility of the raw material matrix [46]. This is the effect of pressure during the osmotic dehydration of fruit tissue with a sucrose solution in place of cell juice, which is more difficult to drain from the material matrix due to drying. In addition, during osmotic dehydration under varied pressure, gas is removed from the fruit tissues, which make up 25% of apples, affecting the pressure inside the pores of the fruit. According to the hydromechanical effect, the osmotic solution penetrating in place of the gas removed increases the surface of the interfacial contact [47], but at the same time has worse thermal properties than the proper cell juice for apple tissue, the properties of which are similar to those of water and are much better than the properties of a hypertonic solution (sucrose 61.5%). The presented arguments may explain the phenomena and the results obtained in this research. The presented images of the structure (Figure 4) and the results of the efficiency of osmotic dehydration (Table 2) testify to the higher concentration of the solution inside the tissue structures.

According to the research carried out on strawberry tissue to determine the effect of fruit processed under the pressure range of 0–250 MPa before drying, Zhang et al. [26] found that pressure stress affected the distribution of moisture in fruit tissue. They observed greater uniformity in tissue hydration in its individual places and found that with increasing pressure during treatment before drying, the availability of water increased, which was the direct cause of reduced vacuum drying and freeze-drying time.

The mechanism of water migration may vary depending on the nature of the raw material being dried and the relationship between the matrix and the amount of moisture that may be removed. There are many descriptions explaining the specificity and dynamics of water movement during drying. One of the mechanisms is the removal of water as a result of capillary interactions, resulting from the difference in concentration, another is surface diffusion using the porous surface theory, describing the diffusion of vapour in pores partially filled with air on the basis of the difference in resilience [1,17,18,48,49,50]. We can also talk about the mechanism of water diffusion under the influence of temperature difference.

Tissue relaxation caused by the action of pressure is an effect of cell walls’ destruction and the partial release of substances from inside the cells that form the structure and maintain the turgor of the plant tissue. The effect is similar to that of blanching [28,51]. At the same time, it is the result of pressure stress, and not a sudden rise in temperature, such as in blanching. Pretreatment with the use of hypertonic solutions carried out at atmospheric pressure and the effects during drying, as well as the properties of drought, resulting from its use with pressure changes (atmospheric—negative pressure, overpressure), pulsed electric field, ultrasound, gamma radiation, high hydrostatic pressures, ohmic heating is confirmed by numerous studies [16,23,25,34,35,37,38,39,40,41,42,43,52,53,54,55]. The properties of dried plant tissue depend not only on the pretreatment but above all on its type, state of ripeness, water content and activity as well as the content of active ingredients at a given moment, which is shown, inter alia, by studies conducted for strawberries [32], apples and bananas [56], apples [19], pomegranate arils [33], goji berry [23], mango [25], persimmon fruit [34,35] and apricot [57].

## 5. Conclusions

The weight variation during osmotic dehydration under varied pressure depends on the process parameters used. The use of lower than and higher than atmospheric pressure during osmotic dehydration results in a reduction in the initial water content. At the same time, the application of varied pressure, regardless of its value, changes the rate of the penetration of the hypertonic solution (osmotic substance) into the tissue of the tested apples, which has an influence on the properties of the final dried product and the drying process. The action of pressure causes the destruction of cell walls and the release of the cell sap. It was found that water diffusivity during drying increased as a result of pressure-induced stress, regardless of its value and the presence of a hypertonic substance in the tissue. By changing the internal structure of osmo-dehydrated and dried apples, the drainage efficiency, defined as the ratio of water loss to solid gain, increases. The structure of apples obtained during osmotic dehydration under negative pressure causes the drying characteristics of materials with a complex capillary–porous structure to be obtained. On the other hand, the course of the drying curves of osmotically dehydrated apples under high pressure is characteristic for materials with a structure similar to clay.

## Figures and Tables

**Table 1:** Process and sample parameters in the obtained dried apples from osmotically dehydrated apples. Mean values marked with the same letter index (a–c) and (A–D) do not differ statistically significantly at the level of *p* = 0.05.

Pressure (MPa)	Final Equilibrium Water Content gH_2_O∙(g d.m.)^−1^	Time Drying (min)
0.02	0.040 ± 0.005 ^C^	250 ± 5 ^a^
0.05	0.039 ± 0.003 ^BC^	250 ± 9 ^a^
0.08	0.041 ± 0.006 ^C^	250 ± 2 ^a^
0.1	0.050 ± 0.001 ^D^	375 ± 2 ^c^
50	0.032 ± 0.003 ^B^	400 ± 2 ^b^
100	0.015 ± 0.004 ^A^	455 ± 5 ^b^
200	0.020 ± 0.007 ^A^	435 ± 5 ^b^
500	0.035 ± 0.004 ^BC^	255 ± 3 ^a^

**Table 2:** Effectiveness of *WL*∙(*SG*)^−1^ osmotic dehydration of apples and increase in dry matter weight in fruit tissue under varied pressure. Mean values marked with the same letter index (a–d) do not differ statistically significantly at the level of *p* = 0.05.

Pressure (MPa)	*WL∙(SG)*^−1^ gH_2_O∙(g d.m.)^−1^	*SG* g d.m.∙(g i.d.m.)^−1^
0.02	1.56 ± 0.17 ^c^	13.24 ± 0.40 ^c^
0.05	2.06 ± 0.39 ^a^	11.18 ± 0.18 ^b^
0.08	2.35 ± 0.38 ^b^	10.16 ± 0.15 ^a^
0.1	2.80 ± 0.43 ^b^	9.91 ± 0.10 ^a^
50	1.37 ± 0.18 ^c^	11.88 ± 1.39 ^bc^
100	2.08 ± 0.06 ^a^	13.41 ± 0.67 ^c^
200	2.19 ± 0.78 ^ab^	13.12 ± 1.40 ^c^
500	2.09 ± 0.26 ^a^	15.27 ± 0.77 ^d^

*WL*—water loss; *SG*—increase in dry matter weight.

## Data Availability

Data of investigations are available from the authors.
